# Generalised free energy and active inference

**DOI:** 10.1007/s00422-019-00805-w

**Published:** 2019-09-27

**Authors:** Thomas Parr, Karl J. Friston

**Affiliations:** Wellcome Centre for Human Neuroimaging, Institute of Neurology, University College London, 12 Queen Square, London, WC1N 3BG UK

**Keywords:** Bayesian, Active inference, Free energy, Data selection, Epistemic value, Intrinsic motivation

## Abstract

Active inference is an approach to understanding behaviour that rests upon the idea that the brain uses an internal generative model to predict incoming sensory data. The fit between this model and data may be improved in two ways. The brain could optimise probabilistic beliefs about the variables in the generative model (i.e. perceptual inference). Alternatively, by acting on the world, it could change the sensory data, such that they are more consistent with the model. This implies a common objective function (variational free energy) for action and perception that scores the fit between an internal model and the world. We compare two free energy functionals for active inference in the framework of Markov decision processes. One of these is a functional of beliefs (i.e. probability distributions) about states and policies, but a function of observations, while the second is a functional of beliefs about all three. In the former (*expected* free energy), prior beliefs about outcomes are not part of the generative model (because they are absorbed into the prior over policies). Conversely, in the second (*generalised* free energy), priors over outcomes become an explicit component of the generative model. When using the free energy function, which is blind to future observations, we equip the generative model with a prior over policies that ensure preferred (i.e. priors over) outcomes are realised. In other words, if we expect to encounter a particular kind of outcome, this lends plausibility to those policies for which this outcome is a consequence. In addition, this formulation ensures that selected policies minimise uncertainty about future outcomes by minimising the free energy expected in the future. When using the free energy functional—that effectively treats future observations as hidden states—we show that policies are inferred or selected that realise prior preferences by minimising the free energy of future expectations. Interestingly, the form of posterior beliefs about policies (and associated belief updating) turns out to be identical under both formulations, but the quantities used to compute them are not.

## Introduction

Over the past years, we have tried to establish active inference (a corollary of the free energy principle) as a relatively straightforward and principled explanation for action, perception and cognition. Active inference can be summarised as self-evidencing (Hohwy 2016), in the sense that action and perception can be cast as maximising Bayesian model evidence, under generative models of the world. When this maximisation uses approximate Bayesian inference, this is equivalent to minimising variational free energy (Friston et al. 2006)—a form of bounded rational behaviour that minimises a variational bound on model evidence. Recently, we have migrated the basic idea from models that generate continuous sensations (like velocity and luminance contrast) (Brown and Friston 2012) to discrete state-space models, specifically Markov decision processes (Friston et al. 2017a). These models represent the world in terms of discrete states, like I am on this page and reading this word (Friston et al. 2017d). Discrete state-space models can be inferred using belief propagation (Yedidia et al. 2005) or variational message passing (Dauwels 2007; Winn 2004) schemes that have a degree of neuronal plausibility (Friston et al. 2017c). The resulting *planning as inference* scheme (Attias 2003; Baker et al. 2009; Botvinick and Toussaint 2012; Verma and Rao 2006) has a pleasingly broad explanatory scope, accounting for a range of phenomena in cognitive neuroscience, active vision and motor control (see Table 1). In this paper, we revisit the role of (expected) free energy in active inference and offer an alternative, simpler and more general formulation. This formulation does not substantially change the message passing or belief updating; however, it provides an interesting perspective on planning as inference and the way that we may perceive the future.

**Table 1:** Applications of active inference for Markov decision processes

Application	Comment	References
Decision making under uncertainty	Initial formulation of active inference for *Markov decision processes* and *sequential policy optimisation*	Friston et al. (2012c)
Optimal control (the mountain car problem)	Illustration of *risk sensitive or KL control* in an engineering benchmark	Friston et al. (2012a)
Evidence accumulation: Urns task	Demonstration of how beliefs states are absorbed into a generative model	FitzGerald et al. (2015b, c)
Addiction	Application to psychopathology	Schwartenbeck et al. (2015c)
Dopaminergic responses	Associating dopamine with the encoding of (expected) precision provides a plausible account of dopaminergic discharges	FitzGerald et al. (2015a), Friston et al. (2014)
Computational fMRI	Using Bayes optimal precision to predict activity in dopaminergic areas	Schwartenbeck et al. (2015a)
Choice preferences and epistemics	Empirical testing of the hypothesis that people prefer to keep options open	Schwartenbeck et al. (2015b)
Behavioural economics and trust games	Examining the effects of prior beliefs about self and others	Moutoussis et al. (2014), Prosser et al. (2018)
Foraging and two-step mazes; navigation in deep mazes	Formulation of epistemic and pragmatic value in terms of *expected free energy*	Friston et al. (2015)
Habit learning, reversal learning and devaluation	Learning as minimising variational free energy with respect to model parameters—and action selection as *Bayesian model averaging*	FitzGerald et al. (2014), Friston et al. (2016)
Saccadic searches and scene construction	*Mean-field approximation* for multifactorial hidden states, enabling high-dimensional beliefs and outcomes, c.f., functional segregation	Friston and Buzsaki (2016), Mirza et al. (2016)
Electrophysiological responses: *place*-*cell activity, omission-related responses, mismatch negativity, P300, phase precession, theta*–*gamma coupling*	Simulating neuronal processing with a gradient descent on variational free energy, c.f., dynamic *Bayesian belief propagation* based on marginal free energy	Friston et al. (2017a)
Structure learning, sleep and insight	Inclusion of parameters into expected free energy to enable structure learning via *Bayesian model reduction*	Friston et al. (2017b)
Narrative construction and reading	Hierarchical generalisation of generative model with *deep temporal structure*	Friston et al. (2017d), Parr and Friston (2017c)
Computational neuropsychology	Simulation of visual neglect, hallucinations and prefrontal syndromes under alternative pathological priors	Benrimoh et al. (2018), Parr and Friston (2017a), Parr et al. (2018a, b, 2019)
Neuromodulation	Use of precision parameters to manipulate exploration during saccadic searches; associating uncertainty with cholinergic and noradrenergic systems	Parr and Friston (2017b, 2019), Sales et al. (2018), Vincent et al. (2019)
Decisions to movements	Hybrid continuous and discrete generative models to implement decisions through movement	Friston et al. (2017c), Parr and Friston (2018)
Planning, navigation and niche construction	Agent-induced changes in environment (generative process); decomposition of goals into subgoals	Bruineberg et al. (2018), Kaplan and Friston (2018)

In current descriptions of active inference, the basic argument goes as follows: active inference is based upon the maximisation of model evidence or minimisation of variational free energy in two complementary ways. First, one can update one’s beliefs about latent or hidden states of the world to make them consistent with observed evidence—or one can actively sample the world to make observations consistent with beliefs about states of the world. The important thing here is that both action and perception are in game of minimising the same quantity, namely variational free energy. A key aspect of this formulation is that action (i.e. behaviour) is absorbed into inference, which means that agents have beliefs about what they are doing—and will do. This calls for prior beliefs about action or policies (i.e. sequences of actions). So where did these prior beliefs come from?

The answer obtains from a *reductio ad absurdum* argument: if action realises prior beliefs and minimises free energy, then the only tenable prior beliefs are that action will minimise free energy. If this were not the case, we reach the following absurd conclusion. If a free energy minimising creature did not have the prior belief that it selects policies that minimise (expected) free energy, it would infer (and therefore pursue) policies that were not free energy minimising. As such, it would not be a free energy minimising creature, which is a contradiction. This leads to the prior belief that I will select policies that minimise the free energy expected under that policy. The endpoint of this argument is that action or *policy selection becomes a form of Bayesian model selection*, where the evidence for a particular policy becomes the free energy expected in the future. This *expected free energy* is a slightly unusual objective function because it scores the evidence for plausible policies based on outcomes that have yet to be observed. This means that the expected free energy becomes the variational free energy expected under (posterior predictive) beliefs about outcomes. These priors are usually informed by prior beliefs about outcomes that play the role of prior preferences or utility functions in reinforcement learning and economics.

In summary, beliefs about states of the world and policies are continuously updated to minimise variational free energy, where posterior beliefs about policies (that prescribe action) are based upon expected free energy (that may or may not include prior preferences over future outcomes). This is the current story and leads to interesting issues that rest on the fact that expected free energy can be decomposed into epistemic and pragmatic parts (Friston et al. 2015). This decomposition provides a principled explanation for the epistemics of planning and inference that underwrite the exploitation and exploration dilemma, novelty, salience and so on. However, there is another way of telling this story that leads to a conceptually different sort of interpretation.

In what follows, we show that the same Bayesian policy (model) selection obtains from minimising variational free energy when *future outcomes are treated as hidden or latent states of the world*. In other words, we can regard active inference as minimising a generalised free energy under generative models that entertain the consequences of (policy-dependent) hidden states of the world in the future. This simple generalisation induces posterior beliefs over future outcomes that now play the role of latent or hidden states. In this setting, the future is treated in exactly the same way as the hidden or unobservable states of the world generating observations in the past. On this view, one gets the expected free energy for free, because the variational free energy involves an expectation under posterior beliefs over future outcomes. In turn, this means that beliefs about states and policies can be simply and uniformly treated as minimising the same (generalised) free energy, without having to invoke any free energy minimising priors over policies.

Technically, this leads to the same form of belief updating and (Bayesian) policy selection but provides a different perspective on the free energy principle per se. This perspective says that self-evidencing and active inference both have one underlying imperative, namely to minimise *generalised free energy* or uncertainty. When this uncertainty is evaluated under models that generate outcomes in the future, future outcomes become hidden states that are only revealed by the passage of time. In this context, outcomes in the past become observations in standard variational inference, while outcomes in the future become posterior beliefs about latent observations that have yet to disclose themselves. In this way, the generalised free energy can be seen as comprising variational free energy contributions from the past and future.

The current paper provides the formal basis for the above arguments. In brief, we will see that both the expected and generalised free energy formulations lead to the same update equations. However, there is a subtle difference. In the expected free energy formalism, prior preferences or beliefs about outcomes are used to specify the prior over policies. In the generalised formulation, prior beliefs about outcomes in the future inform posterior beliefs about the hidden states that cause them. Because of the implicit forward and backward message passing in the belief propagation scheme obtained at the free energy minimum (Yedidia et al. 2005), these prior beliefs or preferences act to distort expected trajectories (into the future) towards preferences in an optimistic way (Sharot et al. 2012). Intuitively, the expected free energy contribution to generalised free energy evaluates the (complexity) cost of this distortion, thereby favouring policies that lead naturally to preferred outcomes—without violating beliefs about state transitions and the (likelihood) mapping between states and outcomes. The implicit coupling between beliefs about the future and current actions means that, in one sense, the future can cause the past.

Framing probabilistic reasoning in terms of inferential message passing formalises several prominent concepts in the study of human decision making. The idea that prior beliefs distort beliefs about future and that this optimism about the future propagates backwards in time to influence behaviour in an adaptive way (McKay and Dennett 2010; Sharot 2011), is highly consistent with an influence of beliefs about the future over beliefs about the present. Simplistically, the idea behind these accounts is that adaptive behaviour relies upon the (possibly false) belief that future events will accord with our preferences. It is only by believing that we will realise these goals that we act in a manner consistent with their realisation. Intuitively, without the belief that we will end up eating dinner, there would be no reason to shop for ingredients. The passing of messages from past to future resonates with the notion that working memory is vital for predicting the future and planning actions accordingly (Gilhooly 2005; Hikosaka et al. 2000), and underwrites research on episodic future thinking and counterfactual reasoning (Schacter et al. 2015). Appealing to bidirectional inferential message passing has enabled us to reproduce a range of behavioural and electrophysiological phenomena through simulation (summarised in Table 1).

This paper comprises three sections. In the first, we outline the approach we have used to date (i.e. minimising the variational free energy under prior beliefs that policies with a low expected free energy are more probable). In the second, we introduce a generalisation of the variational free energy that incorporates beliefs about future outcomes. The third section compares these two approaches conceptually and through illustrative simulations.

## Active inference and variational free energy

The free energy principle is motivated by the defining characteristic of living creatures, namely that they persist in the face of a changing world. In other words, their states occupy a small proportion of all possible states with a high probability. From the perspective of statistical physics, this means that they show a form of self-organised, non-equilibrium steady-state that maintains a low entropy probability distribution over their states. In information theory, self-information or surprise (a.k.a. negative log model evidence) averaged over time is entropy. More generally, entropy is defined in terms of an ensemble average. However, under that assumption that a system has achieved its (non-equilibrium) steady state, the ensemble and time average are equivalent (under mild weakly mixing assumptions). This means, at any given time, all biological systems are compelled to minimise their surprise. While this may seem like a very bold statement, we do not intend to trivialise the many constraints that dictate behaviour. The point here is that when all these constraints are written into a generative model as prior beliefs, they all contribute to the same cost function: surprise. This reframes the problem of expressing the constraints biological systems must satisfy as a problem of specifying the right set of priors. Although the computation of surprise is often intractable, an approximation is simple to calculate. This is variational free energy (Beal 2003; Dayan et al. 1995; Friston 2003) which depends upon specifying a generative model of how data are caused. This generative model comprises a series of conditional probability distributions. For a Markov decision process, it assumes a series of states (*s*) that evolve through time. At each time step, the probability of transitioning from one state to the next depends upon a policy (*π*). Neither states nor policies are directly accessible to the creature in question. However, each state probabilistically generates an observable outcome (*o*). As Jensen’s inequality demonstrates, free energy is an upper bound on surprise.

1 $$ \underbrace {F}_{{{\text{Free}}\,{\text{energy}}}} = \underbrace {{ - E_{{Q(\tilde{s},\pi )}} \left[ {\ln \frac{{P(\tilde{o},\tilde{s},\pi )}}{{Q(\tilde{s},\pi )}}} \right] \ge - \ln E_{{Q(\tilde{s},\pi )}} \left[ {\frac{{P(\tilde{o},\tilde{s},\pi )}}{{Q(\tilde{s},\pi )}}} \right]}}_{{{\text{Jensen}}'{\text{s}}\,{\text{inequality}}}} = \underbrace {{ - \ln P(\tilde{o})}}_{\text{Surprise}} $$

In the equation above, *P* indicates a probability distribution over outcomes $\tilde{o} = (o_{1} ,o_{2} , \ldots ,o_{T} )$ that are generated by hidden states of the world $\tilde{s} = (s_{1} ,s_{2} , \ldots ,s_{T} )$ and policies, which define the generative model. The generative model is thus expressed as a joint probability distribution over outcomes (i.e. consequences) and their causes (i.e. hidden states of the world and policies available to the agent). Marginalising (i.e. summing or integrating) over the states and policies gives the evidence (a.k.a., marginal likelihood). The log of this marginal likelihood is negative surprise. *Q* is a probability distribution over unobservable (hidden) states and policies—that becomes an approximate posterior distribution as free energy is minimised. The minimisation of free energy over time ensures entropy does not increase, thereby enabling biological systems to resist the second law of thermodynamics and their implicit dissipation or decay.

Note that the generative model is not a model of the biological system itself, but an implicit model of how the environment generates its sensory data. The dynamics of inference and behaviour that we are interested in here emerge from minimising free energy under an appropriate choice of generative model. For readers with a physics background, and analogy would be that the free energy plays the role of a Lagrangian whose ‘potential energy’ component is given by the generative model. Just as a Lagrangian is used to recover the equations of motion for a physical system, we use the free energy to recover the belief updates that determine a biological system’s behaviour.

In the following, we begin by describing the form of the generative model we have used to date. We will then address the form of the approximate posterior distribution. To make inference tractable, this generally involves a mean-field approximation that factorises the approximate posterior distribution into independent factors or marginal distributions.

**Fig. 1:**
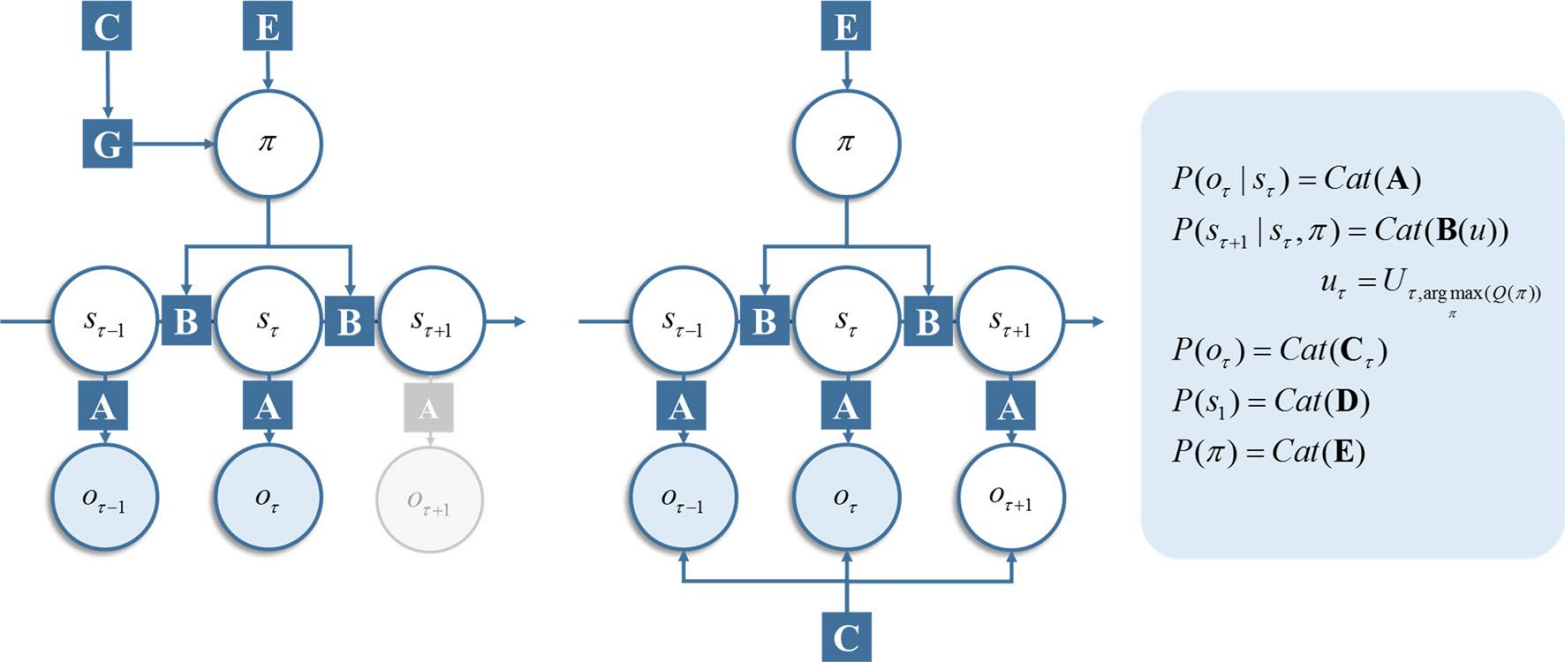
Markov decision process. This shows the basic structure of the discrete state-space generative model used in this paper, assuming the current time is *t *=* τ*. The factor graph *on the left* is the generative model we have used in previous work. Importantly, the prior belief about observations only enters this graph through the expected free energy, $G$ (see main text), which enters the prior over policies. Policies index alternative trajectories, or sequences, of actions. In this sense, they are not time dependent, as each policy determines a sequence of actions for *all* time-points. Conversely, the actions (*u*) are time dependent. *U* is an array that specifies an action for each time step (rows) and each policy (columns). The selected action therefore depends upon the most likely policy and the action that policy implies for that time step. Action selection is technically not part of the generative model, as it relies upon the posterior distribution *Q* (please see main text for details), obtained by inverting the model. This is an important, aspect of active inference, as it underwrites the way in which the system performing inference may change the process generating its observed data. The grey region of this graph indicates that the observation at the next time step is not yet available, so cannot yet be incorporated into the graph. The *right* factor graph is the new version of the generative model considered in this paper. This generative model does not require an expected free energy, and the prior over outcomes enters the model directly as a constraint on outcomes. This also shows a time dependence, as future outcomes are treated as unobserved latent variables (indicated by an unfilled circle). Observed variables are shown as filled circles in both graphs and unobserved variables as unfilled circles. Factors of the generative model (i.e. conditional probability distributions and prior probabilities) are shown as squares. These squares are connected to those circles containing variables that participate in the same factor. Please refer to the main text and Table 2 for a description of the variables. In the panel on the right, the definitions are given for each of the factors in blue squares. Here, Cat refers to the categorical distribution (color figure online)

The generative models used in this paper are subtly different for each free energy functional, but the variables themselves are the same. These are policies ($\pi$) and state trajectories ($\tilde{s}$), all of which are latent (unknown random) variables that have to be inferred. States evolve as a discrete Markov chain, where the transition probabilities are functions of the policy. Likelihood distributions probabilistically map hidden states to observations ($\tilde{o}$). Figure 1 (left) shows these dependencies as a graphical Bayesian network. This type of generative model has been used extensively in simulations of active inference (FitzGerald et al. 2014, 2015c; Friston et al. 2015, 2017a, c, d; Schwartenbeck et al. 2015a), see Table 1.

The role of the generative model is simply to define the free energy functional which, as we will see in Sects. 2.3–2.5, gives rise to the belief update rules that we will employ for our simulations. However, it is helpful to imagine how we might generate data from such a model. We outline this process with the model on the left of Fig. 1 in mind. We could start at the first time step and sample a state from the categorical prior over initial states. The parameters of this prior (**D**) are simply a vector of probabilities for each alternative state. From this, we can now sample from the likelihood. This is formulated as a matrix (**A**), whose columns correspond to a state and whose rows are the alternative outcomes that may be generated. To generate an outcome, we would select the column of this matrix corresponding to the state we sampled and sample an outcome from this column-vector of probabilities. It is this outcome that would be available to a synthetic creature.

Taking a discrete time step into the future, we can sample a new state from the column of a transition matrix (**B**) associated with the state at the previous time. Crucially, the transition probabilities are conditioned upon the selected action. This means we have a separate **B**-matrix for each action. Action selection depends upon the policy, with each policy and time point associated with an action. For the model on the left of Fig. 1, this means we calculate the expected free energy (**G**) for each policy, which depends upon a vector of prior probabilities for outcomes under these policies (**C**). Combining these with a prior bias term (**E**)—as set out in more detail in Sect. 2.4—we can construct a prior over policies. Sampling from this and selecting the action that corresponds to this policy, at this time, specify the **B**-matrix from which to sample the state for the current time step. We could then sample the outcome for this time from the relevant column of the **A**-matrix. This process can be repeated for a series of discrete time steps, generating a new outcome for each time. A similar approach could be taken to generate data from the model on the right of Fig. 1. However, note that the likelihood here comprises both **A** and **C**, and the policy prior only includes **E** (i.e. the expected free energy does not explicitly feature in this model). The procedure outlined above provides an intuition into the beliefs a creature has about how its sensory data are generated by acting on hidden states in the environment.

It is worth noting that the free energy is a *functional* of the distributions in the generative model and of the approximate posterior beliefs, but a *function* of observations. Continuing with this free energy, we now consider the mean-field approximation in current implementations of active inference, and its consequences for the variational free energy. In the next few sections, we unpack the variational free energy, and its role in active inference based on Markov decision processes. The argument that follows is a little involved, but we summarise the key steps here, such that the agenda of each of the following sections is clear. In Sect. 2.1, we specify the form of the variational distribution we employ, and the free energy that results from this. In Sect. 2.2, we unpack the terms in the free energy as they pertain to the generative model. This depends upon having a prior belief about policies. Section 2.3 attempts to identify this prior, through finding the optimal posterior and extrapolating backwards in time. This highlights a shortcoming of this approach that is resolved in Sect. 2.4. In addition to providing a more appropriate prior for policy selection, Sect. 2.4 sets out the role of free energy in simulating behaviour. In brief, this involves finding the variational distribution over policies that minimises free energy. As free energy is a function of sensory observations, this means we need to update these distributions following each new observation. Section 2.5 follows the same approach to find the free energy minima for beliefs about states, giving the fixed points to which these distributions must be updated following each new observation.

### Definition of the mean-field variational free energy

To define the variational free energy for the above generative model, we first need to specify the form of the approximate posterior distribution, $Q$. We do this via a mean-field approximation that treats the (policy dependent) state at each time step as approximately independent of the state at any other time step. We treat the distribution over the policy as a separate factor, which implies a set of (policy) models, $\pi$, over hidden variables $s_{\tau }$:

2 $$ Q(\tilde{s},\pi ) = Q(\pi )\prod\limits_{\tau } {Q(s_{\tau } |\pi )} $$

Mean-field approximations originated in statistical physics, where they can be used to approximate Helmholtz free energy through appealing to an average with respect to a ‘reference’ Hamiltonian. This reference can be simply defined for a system with non-interacting components (or degrees of freedom). In virtue of the assumption that the system’s degrees of freedom do not interact, their Hamiltonian (scaled negative log probability) may be expressed as a sum of contributions from each component. Exponentiating this sum, the probability density can be expressed as a product of marginal probabilities for each degree of freedom. The same idea has been employed extensively in statistical inference and machine learning, where a mean-field approximation refers to the use of a variational distribution comprising a product of marginals (Winn and Bishop 2005; Yedidia et al. 2005). The ‘mean field’ is the expected value of each (log) factor of the generative model (*P*), which include the interactions, under the fully factorised distribution (*Q*). The advantage to using a mean-field approximation is the computational tractability that comes from being able to separately optimise each marginal distribution. We can now substitute this factorised distribution into our definition for the variational free energy above:

3 $$ \begin{aligned} & F = E_{Q(\pi )} [F_{\pi } ] + D_{KL} [Q(\pi )||P(\pi )] \\ & F_{\pi } = - E_{{Q(\tilde{s}|\pi )}} [\ln P(\tilde{o},\tilde{s}|\pi ) - \sum\limits_{\tau } {\ln Q(s_{\tau } |\pi )} ] \\ \end{aligned} $$

In this form, the variational free energy is expressed in terms of policy-dependent terms (second equality) that bound the (negative log) evidence for each policy and a complexity cost or KL divergence^1^ (*D*_KL_) that scores the departure of the posterior beliefs over policies from the corresponding prior beliefs.

### Past and future

There is an important difference in how past and future outcomes are treated by the variational free energy. Note that—as a function of outcomes—the components of the free energy that depend on outcomes can only be evaluated for the past and present. Hidden states, on the other hand, enter the expression as *beliefs* about states. In other words, the free energy is a functional of distributions over states, rather than a function, as in the case of outcomes. This means that free energy evaluation takes account of future states. We can express this explicitly by writing the variational free energy, at time *t*, as a sum over all time steps, factorising the generative distribution according to the conditional independencies expressed in Fig. 1:

4 $$ \begin{aligned} & F_{\pi } = \sum\limits_{\tau } {F_{\pi \tau } } \\ & F_{\pi \tau } = - E_{{Q(s_{\tau } |\pi )Q(s_{\tau - 1} |\pi )}}  \left[ {[\tau \le t] \cdot \ln P(o_{\tau } |s_{\tau } ) + \ln P(s_{\tau } |s_{\tau - 1} ,\pi ) - \ln Q(s_{\tau } |\pi )} \right] \\ \end{aligned} $$

In the above, the Iverson (square) brackets return 1 if the expression is true, and 0 otherwise. It is this condition that differentiates contributions from the past from the future. This equation is obtained in a straightforward way by factorising the generative model (joint distribution) in the second line of Eq. 3, in line with the generative model depicted in Fig. 1. Because the generative model does not include future observations as random variables (given that these data have yet to be collected), there are no accompanying likelihood factors. This reflects the fact that the only data that contribute to the free energy are those we currently have access to. Given the dependence of the right-hand side on the current time (*t*) the free energy should, strictly speaking, be written as a function of *t* and *τ*. As we are interested here in online inference, we will assume an implicit conditioning upon *t* for all free energies throughout this paper. The Iverson brackets above allow us to decompose the sum into past and future components:

5 $$ F_{\pi } = \sum\limits_{\tau \le t} {F_{\pi \tau } + } \underbrace {{\sum\limits_{\tau > t} {E_{{Q(s_{\tau - 1} |\pi )}} [D_{\text{KL}} [Q(s_{\tau } |\pi )||P(s_{\tau } |s_{\tau - 1} ,\pi )]]} }}_{\text{Complexity}} $$

In this decomposition, the contribution of beliefs about future states reduces to a complexity cost. This is the KL divergence between approximate posterior beliefs about states in the future and prior beliefs. The latter are based upon the (policy-specific) transition probabilities in the generative model.

### Policy posteriors and priors

Using the full variational free energy (over all policies) from Eq. 3, we can evaluate posterior beliefs about policies. The variational derivative of the free energy with respect to these beliefs is (where we omit constants, and where $\sigma ( \cdot )$ is a softmax function—i.e. a normalised exponential function):

6 $$ \begin{aligned} & \frac{\delta F}{\delta Q(\pi )} = F_{\pi } - \ln P(\pi ) + \ln Q(\pi ) \\ & \frac{\delta F}{\delta Q(\pi )} = 0 \Leftrightarrow Q(\pi ) = \sigma (\ln P(\pi ) - F_{\pi } ) \\ \end{aligned} $$

The second line derives from the first through rearranging, exponentiating both sides of the equation, and normalising to ensure the approximate posterior sums to one. This, together with Eq. 5, implies the belief prior to any observations (i.e. at $t = 0$), which is given by:

7 $$ Q_{o} (\pi ) = \sigma \left( {\ln P(\pi ) - \sum\limits_{\tau } {E_{{Q(s_{\tau - 1} |\pi )}} \left[ {D_{KL} [Q(s_{\tau } |\pi )||P(s_{\tau } |s_{\tau - 1} ,\pi )]} \right]} } \right) $$

**Fig. 2:**
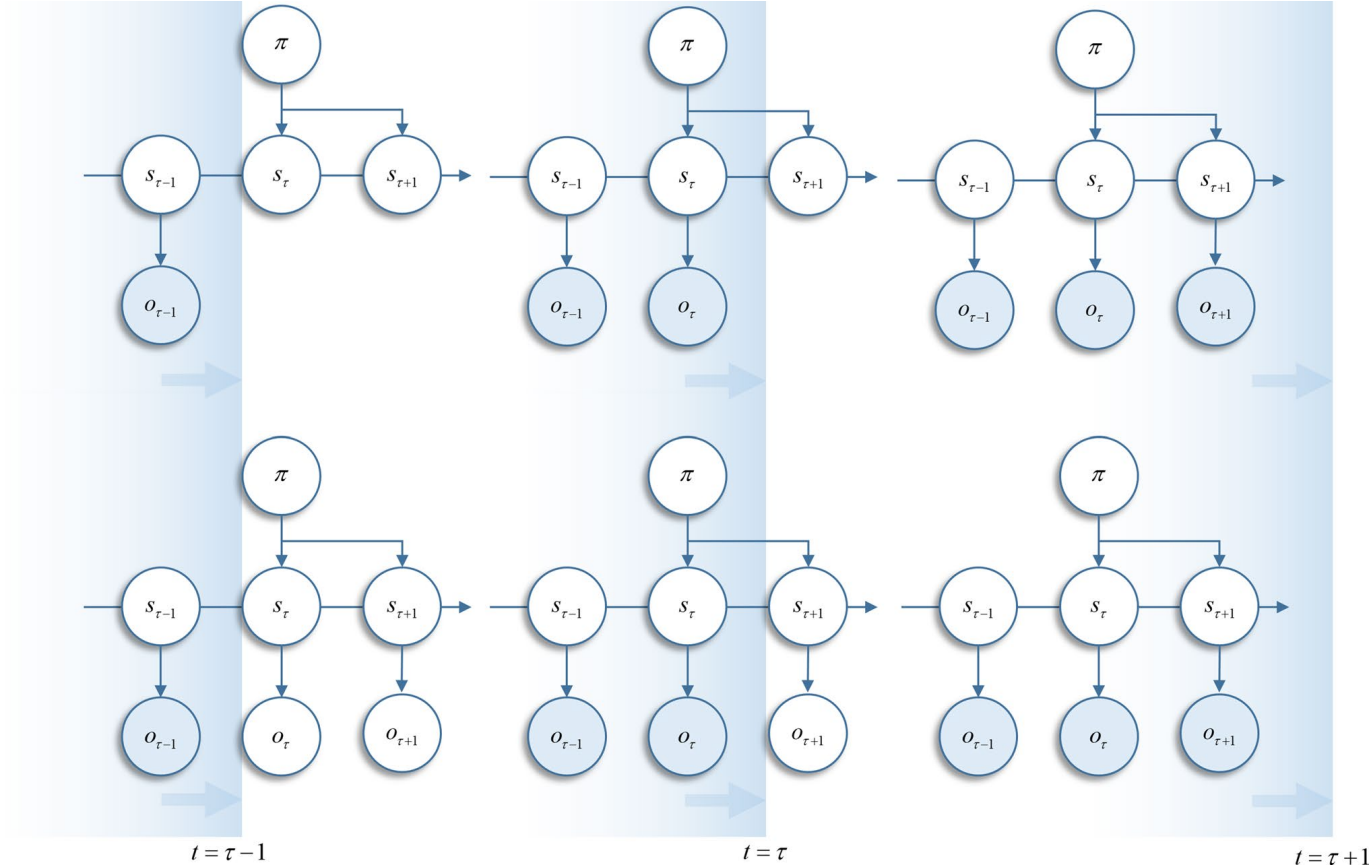
Temporal progression of Markov decision process. The upper graphs shows the structure of the generative model implied using the variational free energy, equipped with a prior that the expected free energy will be minimised by policy selection. Observations are added to the model as they occur. The lower graphs show the structure of the generative model that explicitly represents future outcomes, and minimises a generalised free energy through policy selection. As observations are made, the outcome variables collapse to delta functions. These graphics are intended to highlight two alternative conceptions of a generative model employed in an online setting. The key problem here is how to deal with missing (future) outcomes. These could be omitted until such a time as they become available. Alternatively, they could be treated as hidden variables about which we can hold beliefs. Please note that this graphic illustrates different ways of formulating the generative model used to calculate belief updates. It does not show belief updates, behaviour or any other free energy minimising process. These will be detailed in subsequent sections and figures. However, the reason for making this distinction is important for how we formulate the free energy. The key distinction between the free energies compared in this paper is which of the two perspectives on future outcomes we choose to adopt

This is an unsatisfying result, in which it fails to accommodate our prior knowledge that outcomes will become available in the future. In other words, the posterior at each time step is calculated under a different model (see Fig. 2).

### Expected free energy

To finesse this shortcoming, we can assume agents select the policy that they expect will lead to the lowest free energy (summed over time). This is motivated by the *reductio ad absurdum* in the introduction and is expressed mathematically as:

8 $$ Q_{o} (\pi ) \triangleq \sigma \left( {\ln P(\pi ) - G_{\pi }} \right) $$

This replaces the expression for $Q_{o} (\pi )$ given in Sect. 2.3. (We retain the notation *Q*_*o*_(*π*) for the prior here to distinguish this from the fixed form prior *P*(*π*), which does not depend on the beliefs about states.) *G*_*π*_ is the expected free energy, conditioned on a policy. It is defined as:

9 $$ \begin{aligned} & G_{\pi } = \sum\limits_{\tau > t} {G_{\pi \tau } } \\ & G_{\pi \tau } = - E_{{\tilde{Q}(o_{\tau } ,s_{\tau } |\pi )}} [\ln P(o_{\tau } ,s_{\tau } ) - \ln Q(s_{\tau } |\pi )] \\ \end{aligned} $$

There is an apparent problem with this quantity: The first term within the expectation is a function of outcomes that have yet to be observed. To take this into account, we have defined an (approximate) joint distribution over states and outcomes: $\tilde{Q}(o_{\tau } ,s_{\tau } |\pi ) = P(o_{\tau } |s_{\tau } )Q(s_{\tau } |\pi )$, and take the expectation with respect to this. This means that we can express a (posterior predictive) belief about the observations in the future based on (posterior predictive) beliefs about hidden states. One can obtain a useful form of the expected free energy by rearranging the above: if we factorise the generative model, we obtain:

10 $$ G_{\pi \tau } = - E_{{\tilde{Q}(o_{\tau } ,s_{\tau } |\pi )}} [\underbrace {{\ln P(s_{\tau } |o_{\tau } ) - \ln Q(s_{\tau } |\pi )}}_{{{\text{Epistemic}}\,{\text{value}}}} + \underbrace {{\ln P(o_{\tau } )}}_{{{\text{Extrinsic}}\,{\text{value}}}}] $$

This form shows that policies that have a low expected free energy are those that resolve uncertainty, and that fulfil prior preferences about outcomes. It is the first of these terms that endorses the metaphor of the brain as a scientist, performing experiments (i.e. actions with sensory consequences) to verify or refute hypotheses about the world (Friston et al. 2012b; Gregory 1980). The second term speaks to the notion of a ‘crooked scientist’ (Bruineberg et al. 2016), who designs experiments to confirm prior beliefs, i.e. preferred outcomes. This preference is the same as the evidence (a.k.a., marginal likelihood) associated with a given model. This means policies are selected such that the most probable outcomes under that policy match the most probable outcomes under prior preferences (defined in terms of a marginal likelihood).

Treating $Q(s_{\tau } |\pi )$ as a prior, and $P(s_{\tau } |o_{\tau } )$ as a posterior, we can directly substitute these into Bayes’ rule, which says that their ratio is equal to the ratio of the corresponding likelihood ($Q(o_{\tau } |s_{\tau } ,\pi ) \approx P(o_{\tau } |s_{\tau } )$) and marginal likelihood ($Q(o_{\tau } |\pi )$):

11 $$ \frac{{P(s_{\tau } |o_{\tau } )}}{{Q(s_{\tau } |\pi )}} = \frac{{Q(o_{\tau } |s_{\tau } ,\pi )}}{{Q(o_{\tau } |\pi )}} $$

Due to the symmetry of Bayes’ rule, another perspective on this is that $P(s_{\tau } |o_{\tau } )$ is a likelihood that generates states from observations. This view treats the right-hand side of the above as the ratio between a posterior and a prior. Using this relationship, we can express expected free energy in terms of risk and ambiguity:

$$ G_{\pi \tau } = \underbrace {{D_{KL} [Q(o_{\tau } |\pi )||P(o_{\tau } )]}}_{\text{Risk}} + \underbrace {{E_{{Q(s_{\tau } |\pi )}} [H[\ln P(o_{\tau } |s_{\tau } )]]}}_{\text{Ambiguity}} $$

In this equation, *H* is the Shannon entropy (i.e. negative expected log probability). This means that the prior belief about outcomes enters the generative model through the KL divergence between outcomes expected under any policy and prior preferences. This form also illustrates the correspondence between the expected free energy and the quantities ‘risk’ and ‘ambiguity’ from behavioural economics (Ellsberg 1961; Ghirardato and Marinacci 2002). Risk quantifies the expected cost of a policy as a divergence from preferred outcomes and is sometimes referred to as Bayesian risk or regret (Huggins and Tenenbaum 2015), which underlies KL control and related Bayesian control rules (Kappen et al. 2012; Ortega and Braun 2010; Todorov 2008) and special cases that include Thompson sampling (Lloyd and Leslie 2013; Strens 2000). Ambiguous states are those that have an uncertain mapping to observations. The greater these quantities, the less likely it is that the associated policy will be chosen.

Having identified a suitable prior belief for policies $Q_{o} (\pi )$, we can calculate the fixed point of the free energy with respect to the variational posterior over policies and use this to update the posterior after each time step:

12 $$ \begin{aligned} \frac{\delta F}{\delta Q(\pi )} & = 0 \Leftrightarrow Q(\pi ) = \sigma (\ln Q_{0} (\pi ) - F_{\pi } (\pi )) \\ & = \sigma (\ln P(\pi ) - G(\pi ) - F_{\pi }) \\ \end{aligned} $$

This highlights the way in which the expected free energy influences policy selection. Distributions over policies are updated at each time step to a fixed point that depends upon the expected free energy. The expected free energy is a functional of posterior beliefs about states. Section 2.5 sets out how these may be optimised in relation to sensory outcomes.

### Hidden state updates

To complete our description of active inference, we derive the belief update equations for the hidden states:

13 $$ \begin{aligned} & \frac{{\delta F_{\pi } }}{{\delta Q(s_{\tau } |\pi )}} = - \ln P(o_{\tau } |s_{\tau } ) - E_{{Q(s_{\tau - 1} |\pi )}} [\ln P(s_{\tau } |s_{\tau - 1} ,\pi )] - E_{{Q(s_{\tau + 1} |\pi )}} [\ln P(s_{\tau + 1} |s_{\tau } ,\pi )] + \ln Q(s_{\tau } |\pi ) \\ & \frac{{\delta F_{\pi } }}{{\delta Q(s_{\tau } |\pi )}} = 0 \Leftrightarrow Q(s_{\tau } |\pi ) = \sigma (\ln P(o_{\tau } |s_{\tau } ) + E_{{Q(s_{\tau - 1} |\pi )}} [\ln P(s_{\tau } |s_{\tau - 1} ,\pi )] + E_{{Q(s_{\tau + 1} |\pi )}} [\ln P(s_{\tau + 1} |s_{\tau } ,\pi )]) \\ \end{aligned} $$

**Fig. 3:**
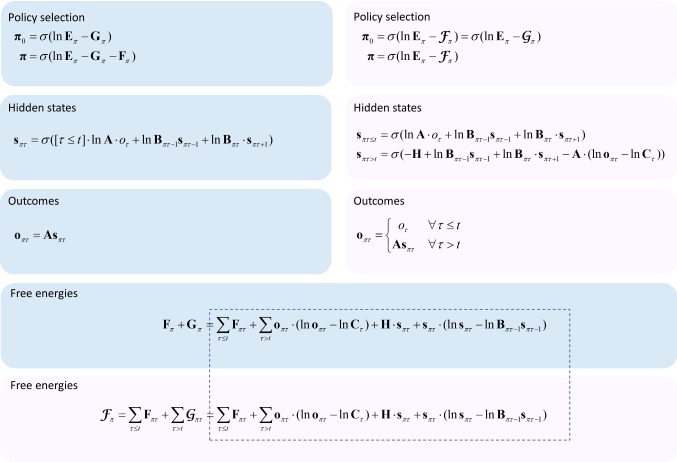
Belief update equations. The blue panels show the update equations using the standard variational approach. The pink panels show the update equations when the generalised free energy is used. The equations in this figure show the fixed points for the sufficient statistics of each variational distribution. These are calculated as in the main text by finding the minima of each of the free energy functionals. As such, updating the variational distributions (left-hand side of each equation) to their fixed points (right-hand side of each equation) following each new observation minimises the corresponding free energy. The dotted outline indicates the correspondence between the generalised free energy and the sum of the variational and expected free energies, and therefore the equivalence of *the form* of the posteriors over policies. However, it should be remembered that the variables within these equations are not identical, as the update equations demonstrate. See Table 2 for the definitions of the variables as they appear here. The equations used here are discrete updates. A more biologically plausible (gradient ascent) scheme is used in the simulations. These simply replace the updates with differential equations that have stationary points corresponding to the variational solutions above. Because the belief updates specified in Fig. 3 take each belief distribution to its free energy minimum, the belief updates and corresponding policy choices necessarily minimise free energy. In the update equations shown here, *o*_*τ*_ is treated as a binary vector with one in the element corresponding to the observed data, and zero for all other elements. This ensures consistency with the linear algebraic expression of the update equations (color figure online)

This result says that, to minimise free energy, we update beliefs about states under policies at each time step such that they are equal to a softmax function of a sum of expected log probabilities. These are the terms in the generative model that depend upon the state about which we optimise beliefs. Technically, these are the state’s Markov blanket (Pearl 1998). These comprise the constraints based upon beliefs about the previous state, the next state and the sensory outcome generated by the current state. The expectations here are simple to calculate, in virtue of the categorical distributions used to define the model and variational posterior. Practically, this means that the sufficient statistics of these are vectors (or matrices, for conditional distributions), where each element is the probability of each alternative value the state can take. (For conditional distributions, these are matrices where each column is a different value for the variable in the conditioning set.) Table 2 sets out the notation used for these sufficient statistics. Crucially, the linear algebraic expression of these statistics means expectations reduce to matrix–vector multiplications or dot products as set out in Fig. 3. Note that as we progress through time, new outcomes become available. As the free energy minima depend upon available outcomes, this means we need to update the variational posteriors following each new outcome.

**Table 2:** Variables in update equations

Variable	Definition
${\mathbf{F}} = \left[ { \ldots ,F_{\pi } , \ldots } \right]^{T}$	Variational free energy
${\mathbf{G}} = [ \ldots ,G_{\pi } , \ldots ]^{T}$	Expected free energy
${\boldsymbol{\mathcal{F}}} = [ \ldots ,\mathcal{F}_{\pi } , \ldots ]^{T}$	Generalised free energy
$$ \begin{aligned} & {\varvec{\uppi}}_{{\mathbf{o}}} ;\,{\varvec{\uppi}}_{{{\mathbf{o}}i}} = Q_{0} (\pi = i) \\ & {\varvec{\uppi}};\,{\varvec{\uppi}}_{i} = Q(\pi = i) \\ \end{aligned} $$	Policy prior and posterior
${\mathbf{s}}_{\pi \tau } ;\,{\mathbf{s}}_{\pi \tau i} = Q(s_{\tau } = i|\pi )$	State belief (for a given policy and time)
${\mathbf{o}}_{\pi \tau } ;\,{\mathbf{o}}_{\pi \tau i} = Q(o_{\tau } = i|\pi )$	Outcome belief (for a given policy and time)
$o_{\tau }$	Outcome
${\mathbf{A}};\,{\mathbf{A}}_{ij} = P(o_{\tau } = i|s_{\tau } = j)$	Likelihood matrix (mapping states to outcomes)
${\mathbf{B}};\,{\mathbf{B}}_{\pi \tau ij} = P(s_{\tau + 1} = i|s_{\tau } = j,\pi )$	Transition matrix (mapping states to states)
${\mathbf{C}};\,{\mathbf{C}}_{\tau i} = P(o_{\tau } = i)$	Outcome prior
${\mathbf{E}};\,{\mathbf{E}}_{i} = P(\pi = i)$	Fixed form policy prior
${\mathbf{H}};\,{\mathbf{H}}_{i} = \sum\limits_{j} {P(o_{\tau } = j|s_{\tau } = i)\ln P(o_{\tau } = j|s_{\tau } = i)}$	Entropy of the likelihood mapping

### Summary

In the above, we have provided an overview of our approach to date. This uses a variational free energy functional to derive belief updates, while policy selection is performed based on an expected free energy. The resulting update equations are shown in Fig. 3 (blue panels). This formulation has been very successful in explaining a range of cognitive functions, as summarised in Table 1. In the following, we present an alternative line of reasoning. As indicated in Fig. 2, there is more than one way to think about the data assimilation and evidence accumulation implicit in this formulation. So far, we have considered the addition of new observations as time progresses. We now consider the case in which (future) outcomes are represented throughout time. This means that future or latent outcomes have the potential to influence beliefs about past states.

## Active inference and generalised free energy

We define the generalised free energy as

14 $$ \begin{aligned} & \mathcal{F} = E_{Q(\pi )} [\mathcal{F}_{\pi } ] + D_{KL} [Q(\pi )||P(\pi )] \\ & \mathcal{F}_{\pi } = \sum\limits_{\tau } {\mathcal{F}_{\pi \tau } } \\ & \mathcal{F}_{\pi \tau } = - E_{{Q(o_{\tau } ,s_{\tau } |\pi )}} [\underbrace {{\ln P(o_{\tau } ,s_{\tau } |s_{\tau - 1} ,\pi )}}_{\text{Energy}} - \underbrace {{\ln Q(o_{\tau } |\pi ) - \ln Q(s_{\tau } |\pi )}}_{\text{Entropy}}] \\ \end{aligned} $$

where, as above, the expectation is with respect to $Q(o_{\tau } ,s_{\tau } |\pi ) = Q(o_{\tau } |s_{\tau } )Q(s_{\tau } |\pi )$. However, we now distinguish the past and the future through the following:

15 $$ Q\left( {o_{\tau } |s_{\tau } } \right) = \left\{ {\begin{array}{*{20}l} {P(o_{\tau } |s_{\tau } )} \hfill & {:\tau > t} \hfill \\ {\delta (o_{\tau } ,o_{\tau }^{*} )} \hfill & {:\tau \le t} \hfill \\ \end{array} } \right. $$

The *δ* here is a Kronecker delta function (a discrete version of a Dirac delta) that is one when the arguments are equal, and zero otherwise. The starred (*) argument indicates the data we have actually observed. In the generalised free energy, the marginals of the joint distribution over outcomes and states define the entropy but the expectation is over the joint distribution. It is important to note that $Q(o_{\tau } ,s_{\tau } |\pi ) \ne Q(o_{\tau } |\pi )Q(s_{\tau } |\pi )$. It is this inequality that underlies the epistemic components of generalised free energy. Interestingly, if we assumed conditional independence between outcomes and hidden states, $Q(o_{\tau } ,s_{\tau } |\pi ) = Q(o_{\tau } |\pi )Q(s_{\tau } |\pi )$, the resulting belief update equations would correspond exactly to a variational message passing algorithm (Dauwels 2007) applied to a model with missing data.

When the expectation is taken with respect to the approximate posteriors, the marginalisation implicit in this definition ensures that

16 $$ \begin{aligned} - E_{{Q(o_{\tau } ,s_{\tau } |\pi )}} [\ln Q(o_{\tau } |\pi )] & = \sum\limits_{{o_{\tau } ,s_{\tau } }} {Q(o_{\tau } ,s_{\tau } |\pi )\ln Q(o_{\tau } |\pi )} \\ & = - \sum\limits_{{o_{\tau } }} {Q(o_{\tau } |\pi )\ln Q(o_{\tau } |\pi )} \\ & = H[Q(o_{\tau } |\pi )] \\ \end{aligned} $$

If we write out the generative model in full and substitute this (omitting constants) into Eq. 14, we can use the same implicit marginalisation to write:

17 $$ \begin{aligned} & \mathcal{F}_{\pi \tau } = - E_{{Q(o_{\tau } |s_{\tau } )Q(s_{\tau } |\pi )}} [\ln P(o_{\tau } |s_{\tau } )] - E_{{Q(s_{\tau } |\pi )Q(s_{\tau - 1} |\pi )}} [\ln P(s_{\tau } |s_{\tau - 1} ,\pi )] \\ & \quad + E_{{Q(o_{\tau } |\pi )}} [\ln Q\left( {o_{\tau } |\pi } \right)] + E_{{Q(s_{\tau } |\pi )}} [\ln Q\left( {s_{\tau } |\pi } \right)] - E_{{Q(o_{\tau } |\pi )}} [\ln P(o_{\tau } )] \\ & Q\left( {o_{\tau } |\pi } \right) = E_{{Q(s_{\tau } |\pi )}} [Q(o_{\tau } |s_{\tau } )] \\ \end{aligned} $$

The implicit generative model now incorporates a prior over observations. This means that the generative model is replaced with that shown on the right of Fig. 1:

18 $$ \begin{aligned} & P\left( {\tilde{o},\tilde{s},\pi |m} \right) = P(\tilde{o}|\tilde{s},m)P(\tilde{s}|\pi )P(\pi ) \\ & P\left( {\tilde{o}|\tilde{s},m} \right) = \tfrac{1}{Z}P(\tilde{o}|\tilde{s})P(\tilde{o}|m) \\ & Z = \sum\limits_{{\tilde{o}}} {P(\tilde{o}|\tilde{s})P(\tilde{o}|m)} \\ \end{aligned} $$

Here, we have defined the distribution over states and observations in terms of two independent factors, a likelihood and a prior over observations, i.e. preferred observations conditioned on the model. For simplicity, we will omit the explicit conditioning on $m$, so that $P(\tilde{o}|m) = P(\tilde{o})$. This quantity plays exactly the same role as that of the preferences in the formulation described in the previous section. However, while it has the same influence over policy selection, it can no longer be interpreted as model evidence. Instead, it is a policy-independent prior that contributes to the evidence.

For past states, this distribution is flat. Crucially, this means the generalised free energy reduces to the variational free energy for outcomes that had been observed in the past. Separating out contributions from the past and the future, we are left with the following:

19 $$ \mathcal{F}_{\pi } = \sum\limits_{\tau \le t} {F_{\pi \tau } } + \sum\limits_{\tau > t} {\mathcal{G}_{\pi \tau } } $$

Unlike $G$ (the expected free energy), $\mathcal{G}$ is the free energy of the expected future. We can rearrange Eq. 17 (for future states) in several ways that offer some intuition for the properties of the generalised free energy.

20 $$ \begin{aligned} \mathcal{G}_{\pi \tau } & = \underbrace {{D_{KL} [Q(s_{\tau } |\pi )||E_{{Q(s_{\tau - 1} |\pi )}} [P(s_{\tau } |s_{\tau - 1} ,\pi )]]}}_{\text{Complexity}} + \underbrace {{D_{KL} [Q(o_{\tau } |\pi )||P(o_{\tau } )]}}_{\text{Risk}} + \underbrace {{E_{{Q(s_{\tau } |\pi )}} [H[P(o_{\tau } |s_{\tau } )]]}}_{\text{Ambiguity}} \\ & = \underbrace {{D_{KL} [Q(s_{\tau } |\pi )||E_{{Q(s_{\tau - 1} |\pi )}} [P(s_{\tau } |s_{\tau - 1} ,\pi )]]}}_{\text{Complexity}} - \underbrace {{D_{KL} [Q(o_{\tau } ,s_{\tau } |\pi )||Q(s_{\tau } |\pi )Q(o_{\tau } |\pi )]}}_{{{\text{Epistemic}}\,{\text{value}} ({\text{Mutual}}\,{\text{information}})}} \\ & \quad - \underbrace {{E_{{Q(o_{\tau } |\pi )}} [\ln P(o_{\tau } )]}}_{{{\text{Extrinsic}}\,{\text{value}}}} \\ \end{aligned} $$

To obtain the mutual information term, we have used the relationship $\ln P(o_{\tau } |s_{\tau } ) = \ln Q(o_{\tau } |s_{\tau } ) = \ln Q(o_{\tau } ,s_{\tau } |\pi ) - \ln Q(s_{\tau } |\pi )$. The imperative to maximise the mutual information (Barlow 1961, 1974; Linsker 1990; Optican and Richmond 1987) can be interpreted as an epistemic drive (Denzler and Brown 2002). This is because policies that (are believed to) result in observations that are highly informative about the hidden states are associated with a lower generalised free energy. As a KL divergence is always greater than or equal to zero, the second equality indicates that the free energy of the expected future is an upper bound on expected surprise.

To find the belief update equations for the policies, we take the variational derivative of the generalised free energy with respect to the posterior over policies and set the result to zero in the usual way:

21 $$ \begin{aligned} \frac{\delta \mathcal{F}}{\delta Q(\pi )} & = \mathcal{F}_{\pi } - \ln P(\pi ) + \ln Q(\pi ) \\ \frac{\delta \mathcal{F}}{\delta Q(\pi )} & = 0 \Leftrightarrow Q(\pi ) = \sigma (\ln P(\pi ) - \mathcal{F}_{\pi } ) \\ \end{aligned} $$

At time $\tau = 0$, no observations have been made, and the distribution above becomes a prior. When this is the case, $\mathcal{F}_{\pi } = \mathcal{G}_{\pi }$, so the prior over policies is:

$$ Q_{o} (\pi ) = \sigma (\ln P(\pi ) - \mathcal{F}_{\pi }^{\tau = 0} ) = \sigma (\ln P(\pi ) - \mathcal{G}(\pi )) $$

If we take the variational derivative of Eq. 17 with respect to the hidden states:

22 $$ \begin{aligned} \frac{{\delta \mathcal{F}_{\pi } }}{{\delta Q(s_{\tau } |\pi )}} & = \ln Q(s_{\tau } |\pi ) - E_{{P(o_{\tau } |s_{\tau } )}} [\ln P(o_{\tau } |s_{\tau } )] \\ & \quad - E_{{Q(s_{\tau - 1} |\pi )}} [\ln P(s_{\tau } |s_{\tau - 1} ,\pi )] - E_{{Q(s_{\tau + 1} |\pi )}} [\ln P(s_{\tau + 1} |s_{\tau } ,\pi )] \\ & \quad + E_{{P(o_{\tau } |s_{\tau } )}} [\ln Q\left( {o_{\tau } |\pi } \right) - \ln P(o_{\tau } )] \\ \frac{{\delta \mathcal{F}_{\pi } }}{{\delta Q(s_{\tau } |\pi )}} & = 0 \Leftrightarrow Q\left( {s_{\tau } |\pi } \right) = \sigma (E_{{P(o_{\tau } |s_{\tau } )}} [\ln P(o_{\tau } |s_{\tau } )] + E_{{Q(s_{\tau - 1} |\pi )}} [\ln P(s_{\tau } |s_{\tau - 1} ,\pi )] \\ & \quad + E_{{Q(s_{\tau + 1} |\pi )}} [\ln P(s_{\tau + 1} |s_{\tau } ,\pi )] \\ & \quad - E_{{P(o_{\tau } |s_{\tau } )}} [\ln Q\left( {o_{\tau } |\pi } \right) - \ln P(o_{\tau } )]) \\ \end{aligned} $$

The derivative of $E_{{Q(o_{\tau } |\pi )}} [\ln Q(o_{\tau } |\pi )]$ is a little complicated, so this is presented step by step in “Appendix B”. The hidden state update has a different interpretation in the past compared to the future:

23 $$ \begin{aligned} & \forall \tau \le t{:}\quad Q\left( {s_{\tau } |\pi } \right) = \sigma (\ln P(o_{\tau } |s_{\tau } ) + E_{{Q(s_{\tau - 1} |\pi )}} [\ln P(s_{\tau } |s_{\tau - 1} ,\pi )] + E_{{Q(s_{\tau + 1} |\pi )}} [\ln P(s_{\tau + 1} |s_{\tau } ,\pi )]) \\ & \forall \tau > t{:}\quad Q\left( {s_{\tau } |\pi } \right) = \sigma ( - H[P(o_{\tau } |s_{\tau } )] + E_{{Q(s_{\tau - 1} |\pi )}} [\ln P(s_{\tau } |s_{\tau - 1} ,\pi )] + E_{{Q(s_{\tau + 1} |\pi )}} [\ln P(s_{\tau + 1} |s_{\tau } ,\pi )] \\ & \quad \quad - E_{{P(o_{\tau } |s_{\tau } )}} [\ln Q\left( {o_{\tau } |\pi } \right) - \ln P(o_{\tau } )]) \\ \end{aligned} $$

The final term for future beliefs implies that future states are considered more probable if they are expected to be similar to those that generate preferred outcomes. In other words, there is an optimistic distortion of beliefs about the trajectory into the future.

### Summary

We have introduced a generalised free energy functional that is expressed as a functional of beliefs about data. The variational free energy can be seen as a special case of this generalised functional, when beliefs about outcomes collapse to delta functions. When we derive update equations (Fig. 3, pink panels) under this functional, the updates look very similar to those based on the variational free energy approach. An important difference between the two approaches is that we have now included the prior probability of outcomes in the generative model. This has no influence over beliefs about the past, but distorts beliefs about the future in an optimistic fashion. This formulation generalises not only the standard active inference formalism, but also active data selection or sensing approaches in machine learning (MacKay 1992) and computational neuroscience (Yang et al. 2016b). See “Appendix A” for a discussion of the relationship between these.

## Comparison of active inference under expected and generalised free energy

The generalised free energy has the appeal that belief updating and policy selection both minimise the same objective function. In contrast, formulations of active inference to date have required two different quantities (the variational free energy and the expected free energy, respectively) to derive these processes. Although the form of belief updating is the same, the belief updates resulting from the use of a generalised free energy are different in subtle ways. In this section, we will explore these differences and show how generalised active inference reproduces the behaviours illustrated in our earlier papers.

The notable differences between the updates are found in the policy prior, the treatment of outcomes and the future hidden state updates. The prior over policies is very similar in both formulations. The expected and generalised free energy (at $\tau = 0$) differ only in that there is an additional complexity term in the latter. This has a negligible influence on behaviour, as the first action is performed *after* observations have been made at the first time step. At this point, the posterior belief about policies is identical, as the variational free energy supplies the missing complexity term. Although the priors are different, in both form and motivation, the posterior beliefs turn out to be computed identically. Any difference in these can be attributed to the quantities used to calculate them, namely the outcomes and the hidden states.

Outcomes in the generalised formulation are represented explicitly as beliefs. This means that the prior over outcomes is incorporated explicitly in the generative model. There are two important consequences of this. The first is that the posterior beliefs about future outcomes (i.e. the probability of future outcomes given those already observed) can be derived in a parsimonious way, without the need to define additional prior distributions. The second is that hidden state beliefs in the future are biased towards these preferred outcomes. A prior belief about an outcome at a particular time point thus distorts the trajectory of hidden states at each time point reaching back to the present. In addition to this, beliefs about hidden states in the future acquire an ‘ambiguity’ term. This means that states associated with an imprecise mapping to sensory outcomes are believed less likely to be inferred. In summary, not only are belief trajectories drawn in optimistic directions, they also tend towards states that offer informative observations.

**Fig. 4:**
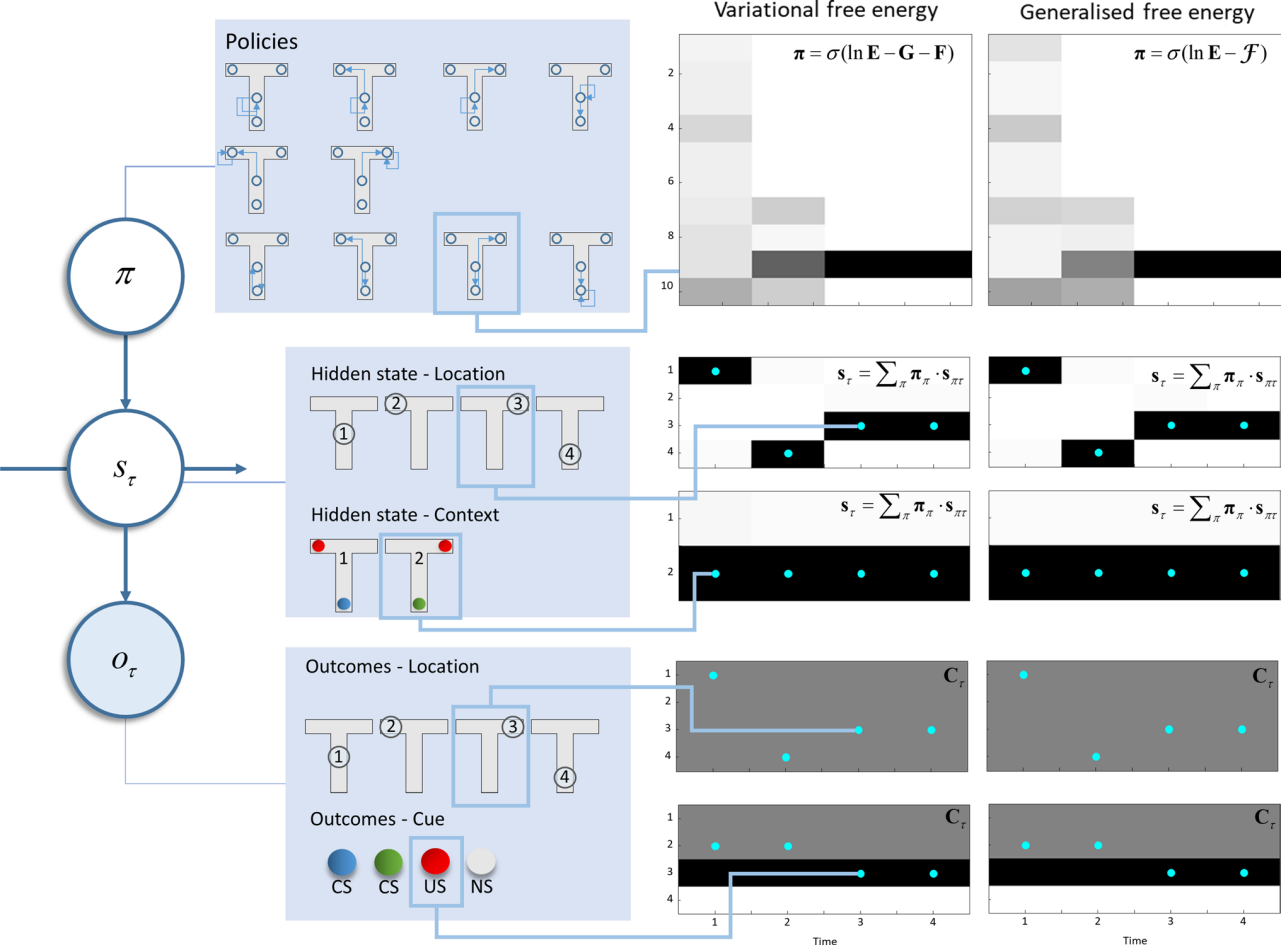
T-maze simulation. The *left* part of this figure shows the structure of the generative model used to illustrate the behavioural consequences of each set of update equations. We have previously used this generative model to address exploration and exploitation in two-step tasks; further details of which can be found in Friston et al. (2015). In brief, an agent can find itself in one of four different locations and can move among these locations. Locations 2 and 3 are absorbing states, so the agent is not able to leave these locations once they have been visited. The initial location is always 1. Policies define the possible sequences of movements the agent can take throughout the trial. For all ten available policies, after the second action, the agent stays where it is. There are two possible contexts: the unconditioned stimulus (US) may be in the left or right arm of the maze. The context and location together give rise to observable outcomes. The first of these is the location, which is obtained through an identity mapping from the hidden state representing location. The second outcome is the cue that is observed. In location 1, a conditioned stimulus (CS) is observed, but there is a 50% chance of observing blue or green, regardless of the context, so this is uninformative (and ambiguous). Location 4 deterministically generates a CS based on the context, so visiting this location resolves uncertainty about the location of the US. The US observation is probabilistically dependent on the context. It is observed with a 90% chance in the left arm in context 1 and a 90% chance in the right arm in context 2. The *right* part of this figure compares an agent that minimises its variational free energy (under the prior belief that it will select policies with a low expected free energy) with an agent that minimises its generalised free energy. The upper plots show the posterior beliefs about policies, where darker shades indicate more probable policies. Below these, the posterior beliefs about states (location and context) are shown, with blue dots superimposed to show the true states used to generate the data. The lower plots show the prior beliefs about outcomes (i.e. preferences), and the true outcomes (blue dots) the agent encountered. Note that a US is preferred to either CS, both of which are preferable to no stimulus (NS). Outcomes are observed at each time step, depending upon actions selected at the previous step. The time steps shown here align with the sequence of events during a trial, such that a new outcome is available at each step. Actions induce transitions from one time step to the next (color figure online)

To make the abstract considerations above a little more concrete, we have employed an established generative model that has previously been used to demonstrate epistemic (i.e. information seeking) behaviours under active inference (Friston et al. 2015). This is a T-maze task (Fig. 4), in which an agent decides between (temporally deep) policies. Temporal depth here refers to the depth of the planning horizon. A temporally deep policy is one that considers sequences of actions, as opposed to only the next action. In one arm, there is an unconditioned^2^ (rewarding) stimulus. In another, there is no stimulus, and this condition is considered aversive. In the final arm, there is always an instructional or conditioned stimulus that indicates the arm that contains the reward. There are two possible contexts for the maze. The first is that where the unconditioned stimulus is in the left arm and the second where it is in the right arm. The starting location and the location of the conditioned stimulus are neither aversive nor rewarding. Under each of the schemes illustrated here, the degree to which a stimulus is rewarding is expressed in terms of the prior preference (i.e. **C**). In other words, we can think of reward as the log probability of a given observation. The more probable an outcome is considered to be, the more attractive it appears to be. This is because policies that do not lead to these outcomes violate prior beliefs and are unlikely to be selected a posteriori. Please see “Appendix A” (term 4) for an interpretation of this that appeals to expected utility theory and risk aversion. There is an important distinction here between schemes based upon Bellman optimality and the scheme on offer here. This is that active inference depends upon probabilistic beliefs and does not assume direct access to knowledge about states of the world. Practically, this means that the agent has no direct access to the hidden states, but must infer them based upon the (observable) outcomes. The importance of this is that the information gain associated with an exploratory behaviour can be quantified by the change in beliefs (or uncertainty reduction) that this behaviour facilitates.

As Fig. 4 shows, regardless of the active inference scheme we use, the agent first samples the unrewarding, but epistemically valuable, uncertainty resolving cue location. This entails moving from the initial location in the centre of the maze, where the agent is uncertain about the context, to the location with the conditioned stimulus. To have made the decision to make this move, the agent updated its beliefs about states of the world (**s**_πτ_) in relation to the outcomes (*o*_1_) available in the central location using the fixed-point solutions shown in the ‘hidden states’ panels of Fig. 3. It does so for beliefs about every time point from the start to the end of the (four step) planning horizon. As these belief updates were derived by finding the free energy minima, this means these belief updates necessarily minimise free energy. Once beliefs have been optimised, they may be used to compute the expected free energy (or the corresponding part of the generalised free energy) as in the ‘free energies’ panel of Fig. 3. These are then used to update beliefs about policies as in the ‘policy selection’ panel. In computing these free energies, we required a posterior predictive belief about outcomes, which can be obtained using the likelihood probabilities to project beliefs about states to beliefs about outcomes (‘outcomes’ panel of Fig. 3). Given that the context unambiguously determines the conditioned stimulus and that our agent is initially uncertain about the context, the greatest information gain (and therefore smallest expected or generalised free energy) is associated with policies that sample this cue location.

On reaching the conditioned stimulus and observing the green conditioned stimulus (*o*_2_), the agent again updates beliefs about states to their new fixed point. Here, the free energy minimum corresponds to the belief that the second context (with the unconditioned stimulus in the right arm) is in play. Having resolved uncertainty about the context of the maze, the agent proceeds to maximise its extrinsic reward by moving to the reward location and finding the unconditioned stimulus (*o*_3_). This is consistent with the smaller expected and generalised free energies associated with policies that realise prior beliefs about outcomes (**C** in the ‘free energies’ panels of Fig. 3).

**Fig. 5:**
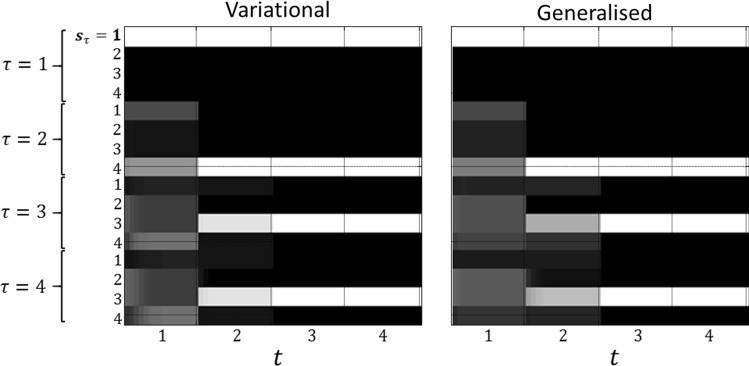
Optimistic distortions of future beliefs. These raster plots represent the (Bayesian model average of the) approximate posterior beliefs about states (specifically, those pertaining to location). At each time step *t*, there is a set of units encoding beliefs about every other time step *τ* in the past and future. The evolution of these beliefs is reflected the evidence accumulation or belief updating of approximate posterior expectations, with lighter shades indicating more probable states

Although the most striking feature of these simulation results is their similarity, there are some interesting differences worth considering. These are primarily revealed by the beliefs about hidden states over time. Under each of the schemes presented here, for a hypothetical rat performing this task, there exist a set of (neuronal) units that encode beliefs about each possible state. For each state, there are units representing the configuration of that state in the past and future, in addition to the present. The activity in these units is shown in Fig. 5. The differences here are more dramatic than in the subsequent behaviours illustrated in Fig. 4. At the first time step (column 1), both agents infer that they will visit location 4 at the next time, resolving uncertainty about the context of the maze. From this future point onwards, however, the beliefs diverge. This can be seen clearly in the lower rows of column 1: the beliefs about the future at the first time step. The agent who employs expected free energy believes they will stay in the uncertainty resolving arm of the maze, while the generalised agent believes they will end up in one of the (potentially) rewarding arms. Despite a shared proximal belief trajectory, the distal elements of the two agents’ paths are pulled in opposite directions. As each future time point approaches, the beliefs about that time begin to converge—as observations become available.

Taken together, Figs. 4 and 5 illustrate an interesting feature of the generalised formulation. Although subtle, at *t *=1, beliefs about location at *τ* = 2 are different, as shown in Fig. 5. Specifically, locations 2 and 3 appear slightly more probable, at the expense of location 4. This illustrates that beliefs about the proximal future are distorted by beliefs about future outcomes. Similarly, at *t *=2, the generalised scheme considers it more likely that it will transition to location 4 relative to the variational scheme. Referring back to Fig. 4, we see that this corresponds to an increased posterior probability for policy 10 at this time step. Here, beliefs about future states and outcomes have influenced beliefs about the plausibility of different behavioural options at the present. In this case, the agent believes that it will experience observations associated with states 2 and 3 in the distal future (*τ* = 4). This enhances the probability of being in states in the more proximal future that are consistent with transitions into states 2 or 3. As these are absorbing states (the probability of staying in those states, once occupied, is one), these states are highly consistent with a transition to themselves. This induces a belief that states 2 and 3 are more probable at time *τ* = 3. Note that, as there are other plausible states that could have transitioned into 2 and 3 at *τ* = 4, the probability of states 2 and 3 at *τ* = 3 is less than at *τ* = 4. The same reasoning explains the higher probability of 2 and 3 at *τ* = 2 (relative to the standard scheme), but with a lower probability relative to occupying these states at later times. If instead the agent believed there was a very low probability of ending up at the goal location, this would induce beliefs that those states that lead to these locations with high probability were themselves unlikely. Another way of putting this is that if I had strong beliefs about where I were to end up, I could infer where I might have been immediately before this. This will depend upon the relative probabilities of going from plausible penultimate locations to the goal location. By propagating these back to the present, I will infer that the most probable trajectory is the one that leads to this goal, and will act to fulfil my beliefs about this trajectory. In the absence of, possibly false, beliefs about where I would end up, I would not end up acting to fulfil these beliefs.

## Conclusion

The generalised free energy introduced in this paper provides a new perspective on active inference. It unifies the imperatives to minimise variational free energy with respect to data, and expected free energy through model selection, under a single objective function. Like the expected free energy, this generalised free energy can be decomposed in several ways, giving rise to familiar information theoretic measures and objective functions in Bayesian reinforcement learning. Generalised free energy minimisation replicates the epistemic and reward seeking behaviours induced in earlier active inference schemes, but prior preferences now induce an optimistic distortion of belief trajectories into the future. This allows beliefs about outcomes in the distal future to influence beliefs about states in the proximal future and present. That these beliefs then drive policy selection suggests that, under the generalised free energy formulation, (beliefs about) the future can indeed cause the past.

## Data Availability

Although the generative model changes from application to application, the belief updates described in this paper are generic and can be implemented using standard routines (here spm_MDP_VB_X.m). These routines are available as MATLAB code in the SPM academic software: http://www.fil.ion.ucl.ac.uk/spm/. Simulations of the sort reported above can be reproduced (and customised) via a graphical user interface by typing in ≫ DEM and selecting the ‘+’ next to the ‘Habit learning’ button.

## Appendix A: Active data selection

Active data selection has been a topic of interest in both neuroscience and machine learning for a number of years (Krause 2008). Several different approaches have been taken to define the best data to sample (Settles 2010), and the optimal experiments to perform to do this (Daunizeau et al. 2011). This appendix addresses the relationship between the future components of the expected free energy and established methods. Writing in full, the (negative) free energy of the expected future is

$$ - G_{\pi \tau } = \underbrace {{H[Q(s_{\tau } |\pi )]}}_{1} + \underbrace {{E_{Q} [\ln P(s_{\tau } |s_{\tau - 1} ,\pi )]}}_{2} + \underbrace {{H[Q(o_{\tau } |\pi )]}}_{3} + \underbrace {{E_{Q} [\ln P(o_{\tau } )]}}_{4} - \underbrace {{E_{Q} [H[P(o_{\tau } |s_{\tau } )]]}}_{5} $$

Under active inference, the above functional is maximised. If we were to use only term 3, this maximisation reduces to ‘uncertainty sampling’ (Hwa 2004; Lewis and Gale 1994; Shewry and Wynn 1987). This involves (as the name suggests) selecting the data points about which uncertainty is highest. A problem with this approach is that it may favour the sampling of ambiguous (uninformative) data. This means that location 1 in our simulation would be very attractive, as there is always a 50% chance of observing each (uninformative) cue. A more sophisticated objective function includes both 3 and 5 (Denzler and Brown 2002; Lindley 1956; MacKay 1992; Yang et al. 2016a). This means that uncertain data points are more likely to be sampled, but only if there is an unambiguous mapping between the latent variable of interest and the data. This renders location 4 more attractive, as it initially associated with uncertain observations but also a precise likelihood mapping. If we were to use just term 5, location 4 would continue to be attractive even after being observed. The contribution of term 3 is to implement a form of ‘inhibition of return’. The relative influences of these terms are unpacked in greater detail in (Parr and Friston 2017b). Term 4 is a homologue of expected utility (reward) in reinforcement learning (Sutton and Barto 1998) and is an important quantity in sequential statistical decision theory (El-Gamal 1991; Wald 1947). On its own, this would not lead to any information seeking behaviour. Terms 1 and 2 together contribute to an ‘Occam factor’ (Rasmussen and Ghahramani 2001), a component of some previously used objective functions (MacKay 1992). We have assumed here that active learning models are myopic, but this is not necessarily the case. On inclusion of an explicit transition model, these models implicitly acquire terms 1 and 2 in the evaluation of posterior beliefs.

Many of these schemes rely upon exact, as opposed to approximate, Bayesian inference. The former can be seen as a special case of the latter, in which the distributions *Q* are equal to the true posteriors. In some cases, this will violate the mean-field assumption used here, and such cases will factorise in a slightly more complicated way. However, it is still possible to represent the terms above in exactly the same way, as long as we introduce an additional corrective (mutual information) term to account for pairwise interactions in the marginal posterior distributions. This is the approach taken in the Bethe free energy that underwrites exact inference procedures such as belief propagation (Pearl 2014; Yedidia et al. 2005).

All of these quantities are emergent properties of a system that minimises its expected free energy. In the schemes mentioned above, the quantities were pragmatically selected to sample data efficiently. Here, they can be seen as special cases of the free energy functional used to define the active inference or sensing that underwrites perception (Friston et al. 2012b; Gregory 1980).

## Appendix B: Variational derivative of expected marginal

Below are the steps taken to obtain the variational derivative of an expected marginal. This is needed for the hidden state update equations under the generalised free energy. For those unfamiliar with variational calculus, “Appendix C” provides a brief introduction.

$$ \begin{aligned} & \frac{\delta }{{\delta Q(s_{\tau } |\pi )}}E_{{Q(o_{\tau } ,s_{\tau } |\pi )}} [\ln Q(o_{\tau } |\pi )] \\ & \quad = E_{{Q(o_{\tau } |s_{\tau } )}} [\ln Q(o_{\tau } |\pi )] + E_{{Q(o_{\tau } ,s'_{\tau } |\pi )}} \left[ {\frac{\delta }{{\delta Q(s_{\tau } |\pi )}}\ln E_{{Q(s_{\tau } |\pi )}} [Q(o_{\tau } |s_{\tau } )]} \right] \\ & \quad = E_{{Q(o_{\tau } |s_{\tau } )}} [\ln Q(o_{\tau } |\pi )] + E_{{Q(o_{\tau } ,s'_{\tau } |\pi )}} \left[ {\frac{{Q(o_{\tau } |s_{\tau } )}}{{E_{{Q(s_{\tau } |\pi )}} [Q(o_{\tau } |s_{\tau } )]}}} \right] \\ & \quad = E_{{Q(o_{\tau } |s_{\tau } )}} [\ln Q(o_{\tau } |\pi )] + \sum\limits_{{o_{\tau } }} {\left[ {\frac{{\sum\nolimits_{{s'_{\tau } }} {Q(o_{\tau } |s_{\tau }^{{\prime }} )} Q(s_{\tau }^{{\prime }} |\pi )}}{{\sum\nolimits_{{s_{\tau } }} {Q(o_{\tau } |s_{\tau } )} Q(s_{\tau } |\pi )}}Q(o_{\tau } |s_{\tau } )} \right]} \\ & \quad = E_{{Q(o_{\tau } |s_{\tau } )}} [\ln Q(o_{\tau } |\pi )] + 1 \\ \end{aligned} $$

In the update equations, we can omit the constant 1.

## Appendix C: A primer on variational calculus

This appendix offers a brief introduction to variational calculus, with a focus on the notion of a functional (or variational) derivative. Variational calculus deals with the problem of finding a function (*f*) that extremises a functional (a function of a function). Formally, this means we try to find:

$$ \begin{aligned} & \tilde{f}(x) = \mathop {\arg \hbox{min} }\limits_{f} S[f(x)] \\ & S[f(x)] \triangleq \sum\limits_{x} {\mathcal{L}(f(x),x)} \\ \end{aligned} $$

We can solve this problem by parameterising our function in terms of a second (arbitrary) function (*g*) and a scalar (*u*):

$$ \begin{aligned} f(x,u) & \triangleq \tilde{f}(x) + ug(x) \\ \frac{\partial }{\partial u}S[f(x,u)] & = \sum\limits_{x} {\frac{\partial }{\partial u}\mathcal{L}(f(x,u),x)} \\ & = \sum\limits_{x} {\frac{\partial f(x,u)}{\partial u}\frac{\partial }{\partial f}\mathcal{L}(f(x,u),x)} \\ & = \sum\limits_{x} {g(x)\frac{\partial }{\partial f}\mathcal{L}(f(x,u),x)} \\ \end{aligned} $$

When *u* is zero, $f = \tilde{f}$ and the functional is minimised. This implies:

$$ \left. {\frac{\partial }{\partial u}S[f(x,u)]} \right|_{u = 0} = 0 = \sum\limits_{x} {g(x)\frac{\partial }{\partial f}\mathcal{L}(f(x),x)} $$

As *g* may be any arbitrary function, the only way that the expression above will always hold is if the partial derivative of $\mathcal{L}$ with respect to *f* is zero for all *x*. This tells us (using *δ* to indicate a variational derivative with respect to a function):

$$ \tilde{f}(x) = \mathop {\arg \hbox{min} }\limits_{f} S[f(x)] \Leftrightarrow \left. {\frac{\delta S}{\delta f}} \right|_{{f = \tilde{f}}} = 0 \Leftrightarrow \left. {\frac{{\partial \mathcal{L}}}{\partial f}} \right|_{{f = \tilde{f}}} = 0 $$

Note that had we assumed that $\mathcal{L}$ was also a function of the gradient of *f*, we would instead have recovered the Euler–Lagrange equation used in analytical mechanics, for which $\mathcal{L}$ is referred to as a Lagrangian. In the main text of this paper, the functional (*S*) is typically a free energy, the function (*f*) is an approximate posterior distribution, and *x* are hidden states. For example:

$$ \begin{aligned} & \mathcal{L}(Q(\tilde{s}|\pi ),\tilde{s}) = Q(\tilde{s}|\pi )(\ln Q(\tilde{s}|\pi ) - \ln P(\tilde{o},\tilde{s}|\pi )) \\ & S = F_{\pi } = \sum\limits_{{\tilde{s}}} {\mathcal{L}(Q(\tilde{s}|\pi ),\tilde{s})} \\ & P(\tilde{s}|\pi ,\tilde{o}) \approx \mathop {\arg \hbox{min} }\limits_{{Q(\tilde{s}|\pi )}} F_{\pi } \\ \end{aligned} $$

This says that, to minimise a free energy functional with respect to a probability distribution, we need to find the point at which the partial derivative of the term inside the sum, with respect to this distribution, is zero.

We hope that this appendix is sufficient for readers not familiar with this style of mathematics to gain some intuition for the variational derivatives used throughout this paper. For interested readers, a more comprehensive introduction to this field can be found in (Moiseiwitsch 2013). For applications in the context of variational inference, please see (Beal 2003).

## Footnotes

1 The KL divergence (also known as relative entropy or information gain) is defined as follows: $D_{\text{KL}} [Q(x)||P(x)] \triangleq E_{Q(x)} [\ln Q(x) - \ln P(x)]$.

2 The terms *conditioned stimulus* and *unconditioned stimulus* are used in the sense of classical (Pavlovian) conditioning paradigms.
